# Autophagosome–lysosome fusion is independent of V-ATPase-mediated acidification

**DOI:** 10.1038/ncomms8007

**Published:** 2015-05-11

**Authors:** Caroline Mauvezin, Péter Nagy, Gábor Juhász, Thomas P. Neufeld

**Affiliations:** 1 Department of Genetics, Cell Biology and Development, University of Minnesota, 6-160 Jackson Hall, 321 Church Street SE, Minneapolis, Minnesota 55455, USA; 2 Department of Anatomy, Cell and Developmental Biology, Eötvös Loránd University, Pazmany s. 1/C. 6.520, Budapest H-1117, Hungary

## Abstract

The ATP-dependent proton pump V-ATPase ensures low intralysosomal pH, which is essential for lysosomal hydrolase activity. Based on studies with the V-ATPase inhibitor BafilomycinA1, lysosomal acidification is also thought to be required for fusion with incoming vesicles from the autophagic and endocytic pathways. Here we show that loss of V-ATPase subunits in the *Drosophila* fat body causes an accumulation of non-functional lysosomes, leading to a block in autophagic flux. However, V-ATPase-deficient lysosomes remain competent to fuse with autophagosomes and endosomes, resulting in a time-dependent formation of giant autolysosomes. In contrast, BafilomycinA1 prevents autophagosome–lysosome fusion in these cells, and this defect is phenocopied by depletion of the Ca^2+^ pump SERCA, a secondary target of this drug. Moreover, activation of SERCA promotes fusion in a BafilomycinA1-sensitive manner. Collectively, our results indicate that lysosomal acidification is not a prerequisite for fusion, and that BafilomycinA1 inhibits fusion independent of its effect on lysosomal pH.

## Abstract (web summary)

BafilomycinA1 is an autophagy inhibitor, presumably owing to its blocking effect on the lysosomal proton pump V-ATPase. Here the authors show that V-ATPase-deficient lysosomes can still fuse with autophagosomes, showing that lysosomal acidification and fusion are two separable, independent events.

## Graphical abstract

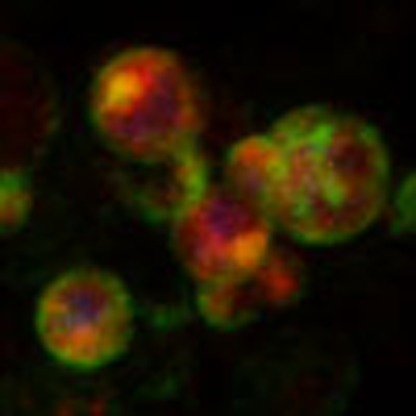

Lysosomal degradation and amino-acid recycling are the ultimate steps of the conserved cellular process known as macroautophagy (hereafter autophagy). As dysregulation of autophagy leads to a wide range of pathologies^1^, a large effort has been made to develop tools for a better understanding and control of the multistep autophagic process^2^. Autophagy begins with formation of an initiating membrane known as the phagophore, which may develop from multiple sources, including ER–mitochondrial junctions, the ER–Golgi intermediate compartment or plasma membrane, leading to formation of the double-membrane autophagosome^3^. This vesicle closes to envelop its target, and then converges and fuses with vesicles from the endocytic pathway, thereby forming amphisomes. Ultimately amphisomes and autophagosomes fuse with lysosomes to form autolysosomes (single-membrane vesicles), whose acidic environment leads to activation of the enzymes essential to degrade biological material.

The V-ATPases are proton pumps that establish and maintain the acidic environment of lysosomes and other membrane-bound compartments by pumping protons into the lumen, a dynamic process that requires ATP hydrolysis. The V-ATPase is a hetero-multimeric enzyme composed of a cytosolic catalytic V1 sector and a membrane-bound V0 proton pore sector. Regulation of the holoenzyme is achieved through reversible binding of the V1–V0 sectors in response to protein kinase A (PKA)-dependent signalling and other cues^4^,^5^. Upon formation of a stable V1–V0 complex, ATP hydrolysis drives rotation of a central stalk domain, facilitating the transfer of two protons across the lysosomal membrane for each molecule of ATP hydrolysed (Supplementary Fig. 1a). V-ATPases promote multiple cellular functions in addition to lysosome-mediated degradation, including sorting of cargo in the endosomal and secretory pathways^6^, proton-coupled transport of ions and solutes and acidification of the pericellular space^7^. Accordingly, perturbation of V-ATPase function has been linked to a broad spectrum of diseases including lysosomal storage disorders, neurodegeneration, myopathy, bone diseases and cancer^8^. A better understanding of V-ATPase function, regulation and pharmacology therefore holds promise of leading to improved disease therapies.

The V-ATPase inhibitor BafilomycinA1 (BafA1) is a macrolide antibiotic derived from *Streptomyces griseus* that targets the V0 sector, inhibiting rotation and passage of protons into the lysosomal lumen, thereby reducing vesicle acidification^9^,^10^. BafA1 also blocks the fusion between autophagosomes and lysosomes in cultured mammalian cells, but the mechanisms are unknown. These dual properties of BafA1 have led to the view that lysosomal acidification is required for fusion. Although regulation of vesicle fusion by V-ATPase proton pump subunits has been described^11^, the specific role of V-ATPase in autophagic vesicle fusion is still unknown.

Here we use genetic analysis in *Drosophila melanogaster* to characterize the relationship between fusion and acidification *in vivo*. In this study, we show that (1) genetic depletion of V-ATPase subunits disrupts lysosomal acidity, blocks autophagic flux and leads to an accumulation of large non-functional autolysosomes; (2) defects in lysosomal acidification do not prevent autophagosome–lysosome fusion; (3) in contrast, BafA1 inhibits autophagosome–lysosome fusion in *Drosophila* as it does in mammalian cells; and (4) BafA1 potentially targets the Ca^2+^ sarco/endoplasmic reticulum Ca^2+^-ATPase (SERCA) pump to inhibit vesicle fusion. These findings thus show that lysosomal acidification and fusion are two separable, independent events. Better understanding of the mode of action of autophagy inhibitors such as BafA1 is essential for further development of such potential therapeutic compounds.

## Results

### Loss of V-ATPase disturbs the autophagic pathway

**Figure 1:**
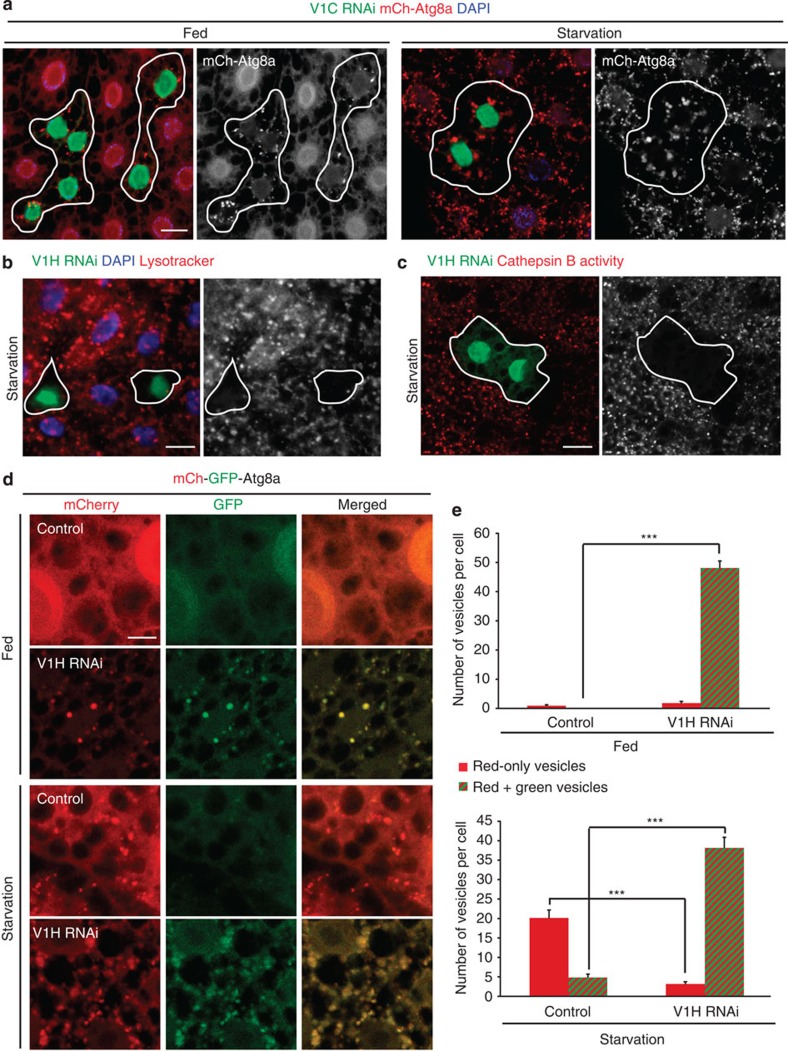
Depletion of V-ATPase subunits leads to accumulation of enlarged, defective autophagic structures. (**a**) Representative confocal images of larval fat body expressing mCh-Atg8a (red), which localizes to autophagosomes and autolysosomes in control cells (GFP negative) under 4 h starvation conditions. Cells expressing the transgenic double-stranded RNA that depletes the V-ATPase subunit *V1C* (GFP-positive clones) accumulate autophagic structures in their cytosol under fed conditions (left) and display enlarged vesicles in starved animals (right). Greyscale images at right show mCh-Atg8a. (**b**) Loss of V-ATPase subunits inhibits vesicle acidification. Fat body cells were stained with LysoTracker Red, which localizes to starvation-induced acidified autolysosomes in control cells (GFP negative). Cells depleted for *V1H* (GFP-positive clones) fail to accumulate acidic organelles. The LysoTracker signal alone is shown in the greyscale image at right. (**c**) Lysosomal enzymes are inhibited in cells depleted for V-ATPase subunits. All cells are stained with Magic Red cresyl violet-(RR)2, which monitors lysosomal Cathepsin B enzyme activity (red). In wild-type cells (GFP negative), Cathepsin B is activated under starvation conditions. Depletion of *V1H* (GFP-positive clone) results in failure to activate Cathepsin B. Magic Red signal alone is shown in greyscale at right. Scale bar, 10 μm. Nuclei are labelled with 4,6-diamidino-2-phenylindole (DAPI; blue) in **a** and **b**. *N*≥6 larvae tested for each genotype or experimental condition. (**d**) Representative confocal images of tandem mCherry-GFP-Atg8a expression in fat body cells. In control animals, starvation leads to the formation of acidic autolysosomes, which can be visualized only in the red channel due to quenching of GFP. In cells depleted for *V1H*, non-acidified (red and green fluorescent) autophagic structures are evident under both nutrient-rich and starvation conditions. Genotypes: control: *Cg-GAL4 UAS-mCherry-GFP-Atg8a/+*. V1H RNAi: *Cg-GAL4 UAS-mCherry-GFP-Atg8a/ UAS-RNAi-V1H*. Scale bar, 5 μm. (**e**) Quantification of number of acidified (red only) and non-acidified (red and green) vesicles from images shown in **d**. Error bars mark s.e.m., *N*≥7 larvae per experimental condition. ***P<0.001, Student’s *t*-test.

The V-ATPase proton pump is an enzymatic macro-complex composed of eight V1, six V0 and two accessory subunits (Supplementary Fig. 1a). V-ATPase structure and composition are well conserved throughout evolution^6^,^12^,^13^. In *Drosophila*, there are 13 and 18 partially redundant orthologues for the V1 and the V0 subunits, respectively (Supplementary Fig. 1a and Supplementary Table 1). The powerful genetics of this system offers a convenient model between yeast and mammalian cells systems to study conserved cellular pathways with low genetic redundancy. We used flippase (FLP)-FLP recognition target (FRT)-mediated recombination to generate genetically mosaic tissue in the larval fat body, which functions similarly to mammalian liver and adipose tissue, and efficiently induces autophagy in response to larval starvation. We first examined the role of V-ATPase subunits in autophagy by characterizing the number and size of structures labelled with mCherry-tagged Atg8a (mCh-Atg8a), a specific marker of autophagosomes and autolysosomes^2^,^14^. To deplete individually each V-ATPase subunit, we tested commercially available RNA interference (RNAi) lines as well as loss of function mutants. Table 1 lists the V-ATPase subunits whose depletion or mutation elicited an autophagy-related phenotype. Surprisingly, depletion of individual V-ATPase subunits led to a highly consistent and robust phenotype independent of subunit size, function or localization within the V-ATPase macro-complex (Table 1), consistent with inactivation of the entire holoenzyme. In V-ATPase-depleted fat body cells, two phenotypes were consistently observed: an inappropriate accumulation of mCh-Atg8a-labelled vesicles in the cells of well-fed animals and a marked increase in vesicle size in response to 4 h starvation (Fig. 1a). These phenotypes were confirmed in V-ATPase loss of function mutant cells using the FRT-FLP system under fed and starved conditions (Supplementary Fig. 1b and Table 1). Given the structural similarities between V- and F-ATPases, we performed similar experiments in cells lacking subunits of the F-ATPase, a related complex responsible for ATP production in mitochondria. Depletion of F-ATPase subunits also led to an abnormal presence of mCh-Atg8a-positive vesicles in the cytosol under nutrient-rich conditions, but vesicle size was not increased by starvation relative to adjacent control cells (Supplementary Fig. 1c). The accumulation of autophagic vesicles under fed conditions in these cells likely reflects ongoing metabolic stress caused by loss of F-ATPase activity and consequently ATP synthesis. Thus, genetic loss of the F-ATPase and V-ATPase leads to distinct cellular responses. In all, loss of V-ATPase induces an accumulation of autophagic structures in the cytosol that expand in size under prolonged stress.

### Vesicle acidification is impaired in V-ATPase-depleted cells

Owing to the robustness and uniformity of the phenotype observed upon knockdown of multiple V-ATPase subunits, we focused our further analysis largely on a single V-ATPase subunit, the regulatory subunit V1H (VhaSFD). V1H was effectively depleted by RNAi (Supplementary Fig. 2d), and this resulted in similar phenotypes whether the RNAi was induced throughout the entire larval fat body tissue or in cell clones (Table 1). V1H depletion had little effect on other cellular parameters, such as cell size, and was phenocopied by a loss of function mutant allele (Table 1 and Supplementary Fig. 1b). First, we aimed to determine whether V1H depletion efficiently inhibited vesicle acidification. In control cells, induction of autophagy leads to formation of autolysosomes that label strongly with LysoTracker, a dye that accumulates in acidic vesicles^15^,^16^. In contrast, cells depleted of V1H did not form LysoTracker-positive structures under either amino-acid starvation (Fig. 1b) or nutrient-rich conditions (Supplementary Fig. 2a). These cells were also deficient for Magic Red staining, a marker for active Cathepsin B, suggesting that the pH within the lysosomal lumen is not acidic enough to support hydrolytic activity during starvation-induced autophagy (Fig. 1c) or under fed conditions (Supplementary Fig. 2b). To further test acidification of autophagic vesicles, we used a tandem-tagged mCherry-GFP-Atg8a construct, which is based on the finding that GFP but not mCherry is quenched upon autophagic delivery of this protein to the acidic environment of the lysosome^17^,^18^. Therefore, only the red mCherry fluorophore is visible in acidic autolysosomes, while both mCherry and GFP remain detectable in defective, non-acidic autolysosomes. In contrast to control cells with acidified autolysosomes, the GFP signal was clearly maintained in cells depleted for V1H, under both fed and starvation conditions (Fig. 1d). Numbers of red-only (acidic) and red+green (yellow, non-acidic) autophagic vesicles were assessed in control and in V1H-depleted cells. Depletion of V1H induced a significant increase in the number of non-acidic autophagosomes in fed and starved cells, and a large decrease in number of acidic autolysosomes under starvation-induced autophagy compared with controls (Fig. 1e). Together, these results strongly suggest that loss of the V-ATPase regulatory subunit V1H blocks autolysosome acidification.

**Figure 2:**
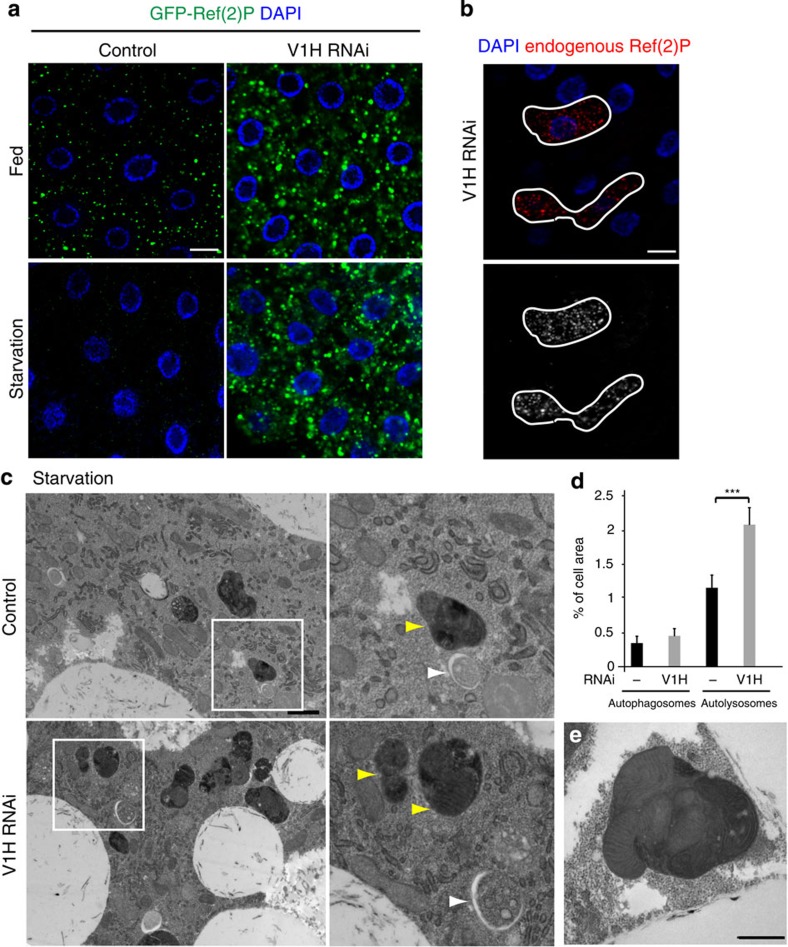
V-ATPase is required for autophagic degradation. (**a**) Representative confocal images showing GFP-Ref(2)P in fat body cells. Starvation-induced autophagy triggers degradation of Ref(2)P-positive aggregates in control animals, whereas Ref(2)P clearance is defective in cells expressing V1H RNAi. *N*≥10 for each genotype. (**b**) Confocal images showing accumulation of endogenous Ref(2)P in cells depleted for the V1H subunit (RNAi-expressing clone is outlined) under nutrient-rich conditions. Endogenous Ref(2)P was detected by immunofluorescence and labelled in red. Ref(2)P signal alone is shown in greyscale. In **a** and **b**, DAPI staining is shown in blue; scale bar, 10 μm. (**c**) TEM images of control (*Cg-GAL4/+*) and V1H-depleted (*Cg-GAL4/UAS-V1H-dsRNA*) fat body cells under starved conditions (4 h). Insets highlight individual autolysosomes (yellow arrowheads) and autophagosomes (white arrowheads). Scale bar, 1 μm. (**d**) Quantification of per cent cell area occupied by double-membrane autophagosomes and single-membrane autolysosomes in control and V1H-depleted cells under starvation conditions. Error bars mark s.e.m., *N*=3 animals, 15 images analysed for each genotype. ***P<0.001, Student’s *t*-test. (**e**) High-magnification TEM image of an enlarged autolysosome from V1H-depleted fat body. Note the intact mitochondria visible within this vesicle. Scale bar, 1 μm.

If autolysosomal acidification is defective and luminal enzymes are inactive upon V-ATPase subunit depletion, we would expect to observe a defect in autophagic cargo degradation. Ref(2)P is a p62 homologue in *Drosophila* that is specifically degraded through autophagy during stress conditions such as amino-acid starvation^19^,^20^,^21^,^22^. Levels of both GFP-tagged and endogenous Ref(2)P were markedly increased in V1H-depleted cells (Fig. 2a,b), confirming that autophagic cargo clearance is defective. Similar results were obtained in cells depleted for the V0 sector subunit V0c (Supplementary Fig. 2c,d).

Ultrastructural analysis further confirmed an abnormal accumulation of autophagic vesicles in cells depleted for V1H, as the percentage of cell area occupied by autolysosomes (identified as single-membrane vesicles containing electron-dense or partially degraded cytoplasmic material) in V1H-depleted cells was approximately twice that of controls (Fig. 2c,d). Furthermore, organelles and cytoplasmic components in V1H-depleted autolysosomes showed less evidence of degradation than in controls, with intact mitochondria visible even after 24 h of starvation (Fig. 2c,e). In contrast, the per cent cell area of double-membrane autophagosomes was not significantly changed in response to V1H depletion. Depletion of the V0c subunit led to an even greater accumulation of autolysosomes (Supplementary Fig. 2e,f). Many of these vesicles appeared swollen and electron lucent, as previously observed in cells treated with basic amines to disrupt lysosomal pH^23^.

Finally, we asked whether reducing lysosomal proteolysis by depleting individual Cathepsin genes could phenocopy depletion of V-ATPase subunits. Surprisingly, this was not the case, as none of the double-stranded RNAs tested against each class of Cathepsin mimicked the effects of V-ATPase depletion, possibly reflecting redundancy owing to the presence of multiple Cathepsin genes (Supplementary Table 2). To summarize, our results demonstrate that the V-ATPase is necessary for autolysosomal acidification and proper autophagic cargo degradation.

### V-ATPase is not required for autophagosome–lysosome fusion

**Figure 3:**
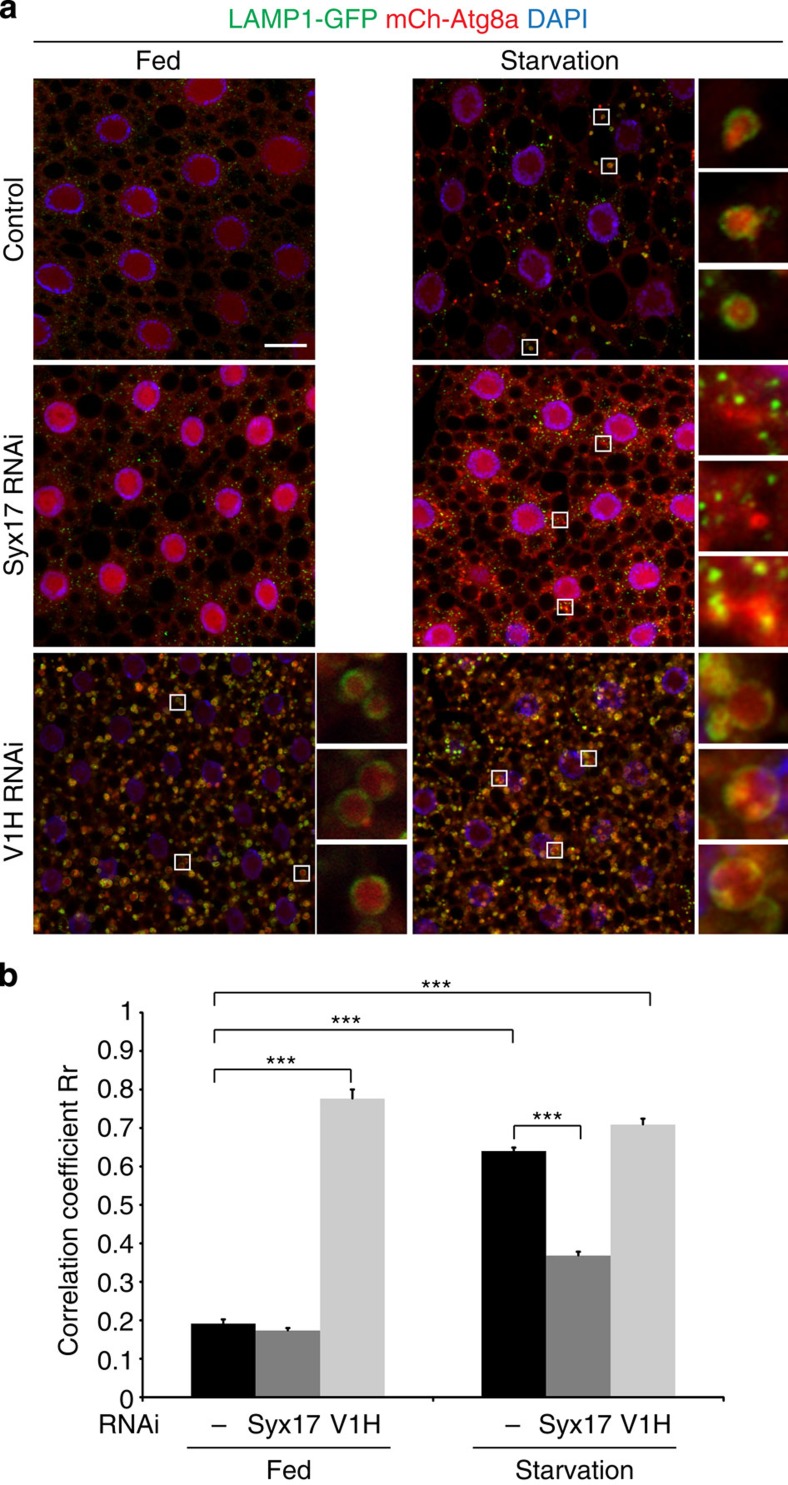
Autophagosome–lysosome fusion is active in V-ATPase-depleted cells. (**a**) Confocal images of larval fat body cells co-expressing mCherry-Atg8a (red), the lysosomal marker LAMP1-GFP (green) and the indicated RNAi constructs under fed or 4 h starved conditions. Insets illustrate the presence of autolysosomes indicating fusion between autophagosomes and lysosomes. Autolysosomes can be identified as mCh-Atg8a-positive structures surrounded by LAMP1-GFP in starved controls and fed or starved V1H-depleted cells. Genotypes: control: *Cg-GAL4 UAS-LAMP1-GFP UAS-mCh-Atg8a/+*. Syx17 RNAi: *Cg-GAL4 UAS-LAMP1-GFP UAS-mCh-Atg8a/ UAS-Syx17-dsRNA*. V1H RNAi: *Cg-GAL4 UAS-LAMP1-GFP UAS-mCh-Atg8a/ UAS-V1H-dsRNA*. Nuclei are labelled in blue with DAPI; scale bars, 10 μm. (**b**) Quantitation of LAMP1-GFP and mCh-Atg8a co-localization as in **a**. Pearson’s correlation coefficient Rr was calculated using ≥10 independent samples for each genotype; error bars mark s.e.m. ***P<0.001, Student’s *t*-test.

BafA1, a chemical inhibitor of the V-ATPase, is commonly used to block autophagosome–lysosome fusion in mammalian cell culture studies, and causes an accumulation of autophagosomes upon autophagy induction^14^,^24^. This phenomenon is reversible, as autolysosomes readily form after washout of BafA1. However, the relevant mechanisms driving the fusion event are unknown, and BafA1 has been shown to have additional targets and effects^25^,^26^. We therefore asked whether the capacity of autolysosomes to properly acidify is required for autophagosome–lysosome fusion *in vivo*. To this end, we examined the co-localization of mCh-Atg8a and the lysosomal marker LAMP1 (lysosomal-associated membrane protein 1)-GFP in control and V1H-depeleted fat body cells, and we quantified these effects by measuring the correlation coefficient (Pearson Rr) under each condition. As an additional control, we depleted the Q-SNARE Syntaxin 17 (Syx17), which is required for autophagosome–lysosome fusion in both *Drosophila* and mammalian cells^27^,^28^. Indeed, cells lacking Syx17 displayed a strong block of autophagic vesicle fusion, accumulating discrete mCh-Atg8a and LAMP1-GFP punctae (Fig. 3a). Induction of autophagy by starvation led to a significant increase in co-localization of mCh-Atg8a and LAMP1-GFP in control animals, which was strongly reduced upon Syx17 depletion (Fig. 3b). In contrast, depletion of V1H did not cause an observable defect in fusion. Enlarged mCh-Atg8a-positive autolysosomes decorated by a well-defined LAMP1-GFP-positive ring were readily detected in cells depleted for V1H (insets in Fig. 3a), and the correlation coefficient between mCh-Atg8a and LAMP1-GFP was significantly higher than controls (Fig. 3b). Significant Atg8a-LAMP1 co-localization was observed under both fed and starved conditions in these cells (Fig. 3a,b), consistent with the abnormal accumulation of autophagic structures detectable in nutrient-rich conditions (Fig. 1a). To determine whether this phenotype is specific to loss of V1H, we depleted additional V-ATPase subunits (V0d and V1D) and again observed Atg8a-LAMP1 co-localization in these cells (Supplementary Fig. 3), suggesting that V-ATPase depletion does not lead to a defect in autophagosome–lysosome fusion.

**Figure 4:**
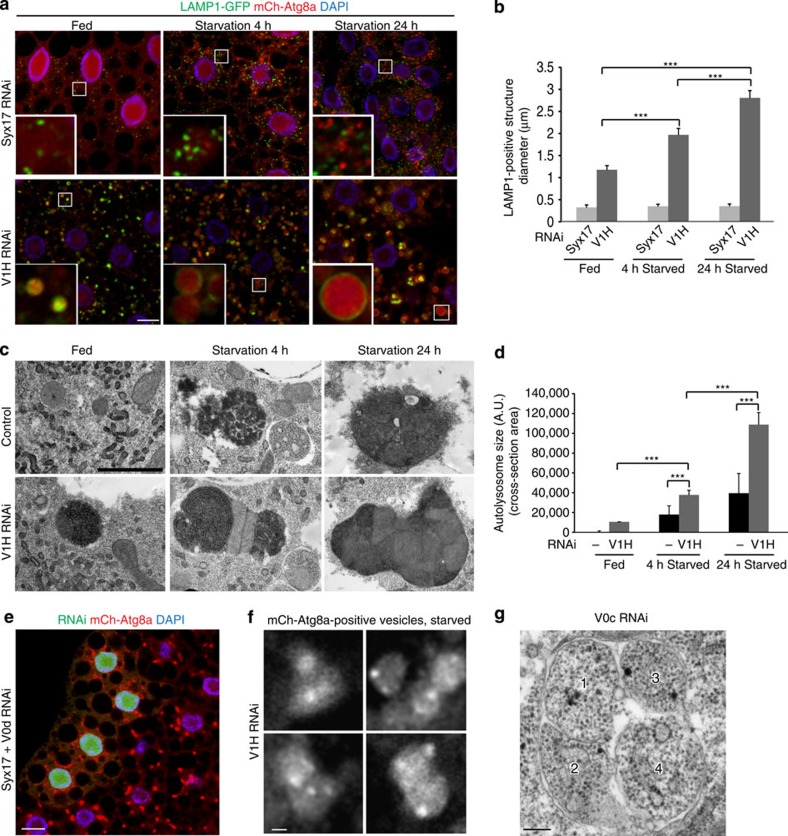
Autophagic vesicles expand under prolonged starvation in V-ATPase-depleted cells. (**a**) Representative images of LAMP1-GFP and mCh-Atg8a-positive vesicles in fat body cells depleted for Syx17 or V1H under fed, 4 h starvation or 24 h starvation conditions. Autophagic vesicles progressively expand in V-ATPase-depleted but not Syx17-depleted cells. (**b**) Quantification of LAMP1-positive vesicle diameter in cells depleted for Syx17 or V1H as shown in **a**. Error bars mark s.e.m.; fat body cells from a minimum of eight larvae were analysed for each genotype and time point. ***P<0.001, Student’s *t*-test. (**c**) TEM images showing representative autolysosomes in control and V1H-depleted fat body cells following 0, 4 and 24 h starvation. Scale bar, 1 μm. (**d**) Quantification of individual autolysosome area in control and V1H-depleted fat body cells as shown in **c**. Vesicles from 15 sections from three larvae were analysed for each genotype and time point. Error bars mark s.e.m.; ***P<0.001, Student’s *t*-test. (**e**) Mosaic fat body containing a GFP-marked clone of cells depleted for Syx17 and the V-ATPase subunit V0d, following 4 h starvation. mCh-Atg8a-marked autophagic vesicles in these cells are not enlarged relative to those in the surrounding GFP-negative control cells. *N*=7. In **a** and **e**, nuclei are labelled in blue with DAPI; scale bars, 10 μm. (**f**) High-magnification confocal images of mCh-Atg8a-positive vesicles in fat body cells depleted of V1H under starvation-induced autophagy (4 h). Variation of fluorescence intensity is observable within individual single vesicles. *N*≥10. Scale bar, 1 μm. (**g**) High-magnification TEM image illustrates a non-degradative autolysosome containing four autophagic bodies (1–4) in V0c-depleted fat body. Scale bar, 0.3 μm.

As lysosomal degradation and turnover is compromised in V-ATPase-depleted cells, ongoing autophagosome–lysosome fusion would be expected to lead to a time-dependent increase in autolysosomal size under autophagic conditions. Indeed, whereas autophagosomes and lysosomes remained small over a 24-h starvation time course in Syx17-depleted cells, we observed a time-dependent increase in autolysosomal size in cells depleted of V1H (Fig. 4a). We quantified this autolysosome expansion phenotype by measuring the diameter of these vesicles. Vesicles marked by mCh-Atg8a and LAMP1-GFP showed a significant expansion following 4 and 24 h amino-acid starvation (1.7- and 2.4-fold diameter increase, respectively) when V1H was depleted throughout the larval fat body (Fig. 4b). This effect was even greater in response to clonal depletion of this subunit, with autolysosomes growing to several micrometre in diameter (Supplementary Fig. 4a,b). Similarly, a significant increase of autolysosome area was observed by ultrastructural analysis of V1H-depleted cells during 24 h starvation (Fig. 4c,d).

The time-dependent expansion of autolysosomes in V-ATPase-depleted cells implies that vesicle fusion is ongoing and active. Indeed, vesicle growth was not observed in cells in which both the V-ATPase and Syx17 were simultaneously depleted (Fig. 4e), indicating that this process reflects autophagosome–lysosome fusion in V-ATPase-depleted cells. Interestingly, multiple mCh-Atg8a-positive spots could often be observed within single LAMP1-GFP vesicles following V1H depletion, presumably resulting from a series of fusion events (Fig. 4f). Analogous structures were identified in transmission electron microscopy (TEM) images of V0c-depleted cells, which displayed large autophagic structures containing multiple intact vesicles with non-digested cytoplasm (Fig. 4g), each presumably resulting from fusion of a single autophagosome with a growing non-functional autolysosome. Note that such autophagic bodies can be routinely visualized in yeast if vacuolar degradation is compromised^29^,^30^, but not in animal cells that contain multiple small lysosomes instead of a single large vacuole.

We examined whether the endocytic pathway also contributes to this vesicle expansion. Interestingly, the endocytic tracer TR-Avidin was internalized normally in V-ATPase-depleted cells, indicating active endocytosis (Supplementary Fig. 4c). Amphisome formation was also not inhibited in cells depleted for V-ATPase, as co-localization of mCh-Atg8a and Rab7-GFP implied normal fusion between autophagosomes and late endosomes (Supplementary Fig. 4d). In addition, successful delivery of a GFP-tagged membrane-bound V0 accessory subunit (VhaM8-9-GFP) to autolysosomes indicates that vesicles emerging from the Golgi apparatus are also able to fuse with lysosomes in V-ATPase-depleted cells (Supplementary Fig. 4e). These results suggest that multiple vesicle pathways converge normally to the lysosomal compartment in V-ATPase-depleted cells, each potentially contributing to autolysosome expansion. Altogether, these findings indicate that vesicle fusion is active in V-ATPase-depleted cells, and, together with a defect in lysosomal degradation, this leads to formation of gigantic non-functional autolysosomes.

### BafA1 inhibits autophagosome–lysosome fusion in *Drosophila*

BafA1 binds to the V0c subunit of the V-ATPase macro-complex and consequently inhibits lysosomal acidification by preventing the translocation of protons across the lysosomal membrane^9^,^31^. BafA1 also induces a block in vesicle fusion and autolysosome formation, and is commonly used in mammalian cell culture to inhibit autophagic flux^14^,^32^. These results have been interpreted to indicate that BafA1 inhibits fusion through its effects on lysosomal acidification. However, our findings demonstrate that lysosomal fusion is not inhibited in response to genetic depletion of V-ATPase subunits in *Drosophila*. One possible explanation for this discrepancy is that lysosomal pH may affect fusion in fundamentally different ways in *Drosophila* and mammalian cells. Alternatively, the effects of BafA1 on fusion may be independent of its effects on acidification.

**Figure 5:**
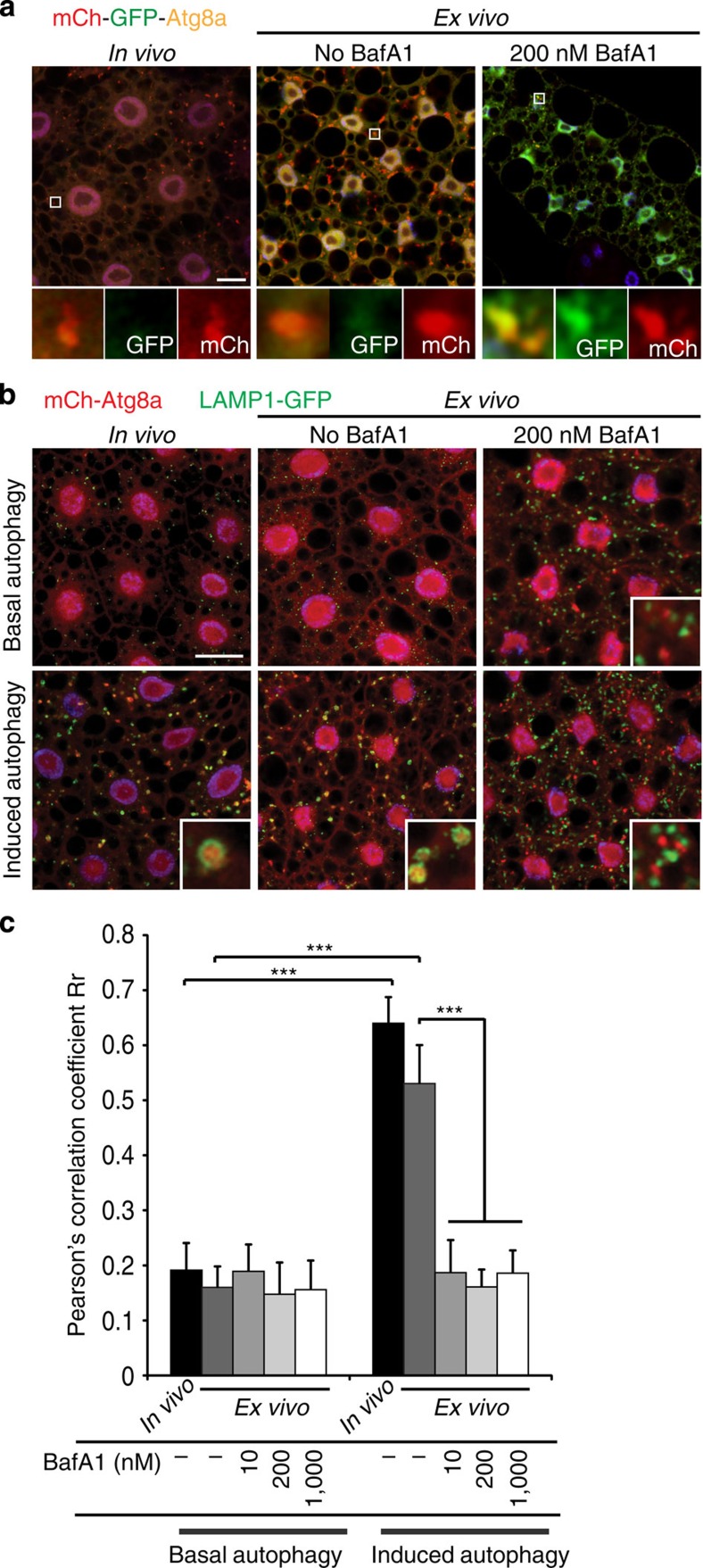
BafA1 inhibits autolysosomal acidification and fusion. (**a**) Representative confocal images of fat body cells expressing the tandem-tagged mCherry-GFP-Atg8a reporter. Following 4 h starvation *in vivo* or 6 h incubation in the absence of BafA1 *ex vivo*, most mCherry-positive punctae exhibit quenching of the GFP signal, indicating its delivery to the acidic lumen of the lysosome. Cells treated with 200 nM BafA1 accumulate non-acidified autophagic structures positive for both GFP and mCherry. Nuclei are labelled with DAPI; blue. *N*≥15 for each genotype. Scale bar, 10 μm. Genotype: *Cg-GAL4 UAS-mCherry-GFP-Atg8a/+*. (**b**) Representative images of fat body cells co-expressing LAMP1-GFP and mCh-Atg8a incubated *ex vivo* for 6 h with 200 nM BafA1 in the presence (basal autophagy) or absence (induced autophagy) of 10 μg ml^−1^ insulin. Nuclei are labelled with DAPI (blue). *N*≥15 for each condition. Scale bar, 10 μm. Genotype: *Cg-GAL4 UAS-mCherry-Atg8a UAS-LAMP1-GFP/+*. (**c**) Quantification of co-localization between LAMP1-GFP and mCh-Atg8a as measured by Pearson’s correlation coefficient Rr, using a minimum of 15 images for each condition. Error bars mark s.d. ***P<0.001, Student’s *t*-test.

To address these issues, we first asked whether BafA1 inhibits autophagic flux in the *Drosophila* larval fat body. Autophagy can be induced *ex vivo* by incubation of fat body in M3 insect cell culture media, an effect that is inhibited by addition of insulin to the media^33^. In control experiments, fat body expressing the tandem-tagged mCherry-GFP-Atg8a displayed quenching of the GFP signal, consistent with active autolysosome acidification under these conditions (Fig. 5a). In contrast, Atg8a punctae remained GFP positive when BafA1 was included in the medium, indicating a defect in acidification or fusion in *ex vivo* BafA1-treated cells compared with control cells (Fig. 5a). Punctate LysoTracker staining in response to autophagy induction was strongly reduced in fat body cells treated with BafA1 (Supplementary Fig. 5a), consistent with impaired lysosome acidification. Thus, both genetic and chemical inhibition of V-ATPase cause a defect in autolysosome acidification.

To determine the effect of BafA1 on autophagosome–lysosome fusion, we examined the co-localization of autophagosome (mCh-Atg8a) and lysosome (LAMP1-GFP) markers in response to BafA1 concentrations of 10 nM, 200 nM (concentration commonly used in mammalian cell culture) and 1 μM. In control experiments, mCh-Atg8a and LAMP1-GFP showed significant co-localization under autophagy-inducing conditions both *ex vivo* and *in vivo*, with Pearson’s correlation coefficients above 0.5 in both cases (Fig. 5b,c). In contrast, each concentration of BafA1 tested led to accumulation of discrete mCh-Atg8a and LAMP1-GFP punctae and reduced the correlation coefficients of these markers to below 0.2 (Fig. 5b,c and Supplementary Fig. 5b). Individual autophagosomes and lysosomes accumulated in fat bodies treated with BafA1 even under non-inducing conditions (Fig. 5b), demonstrating that basal autophagic flux is blocked. Thus, in contrast to the effects of genetic depletion of V-ATPase subunits, BafA1 significantly disrupts autophagosome–lysosome fusion.

### BafA1-driven fusion block is phenocopied by loss of SERCA

The above results indicate that both V-ATPase depletion and BafA1 inhibit vesicle acidification, but only BafA1 blocks autophagosome–lysosome fusion. Taken together, these results imply that BafA1 may affect vesicle fusion through a V-ATPase-independent target. Interestingly, BafA1 has been shown to also inhibit the P-type ATPases (E1-E2-ATPase pumps) with moderate efficiency^26^. In particular, SERCA shows an intermediate sensitivity to BafA1^34^. SERCA is responsible for transporting Ca^2+^ into the ER lumen^35^, which can influence vesicle fusion and autophagosome formation^36^. Thapsigargin, a specific inhibitor of SERCA^37^, was recently shown to block autophagosome–endosome fusion and to inhibit recruitment of the late endosome/lysosome marker Rab7 to autophagosomes^38^.

**Figure 6:**
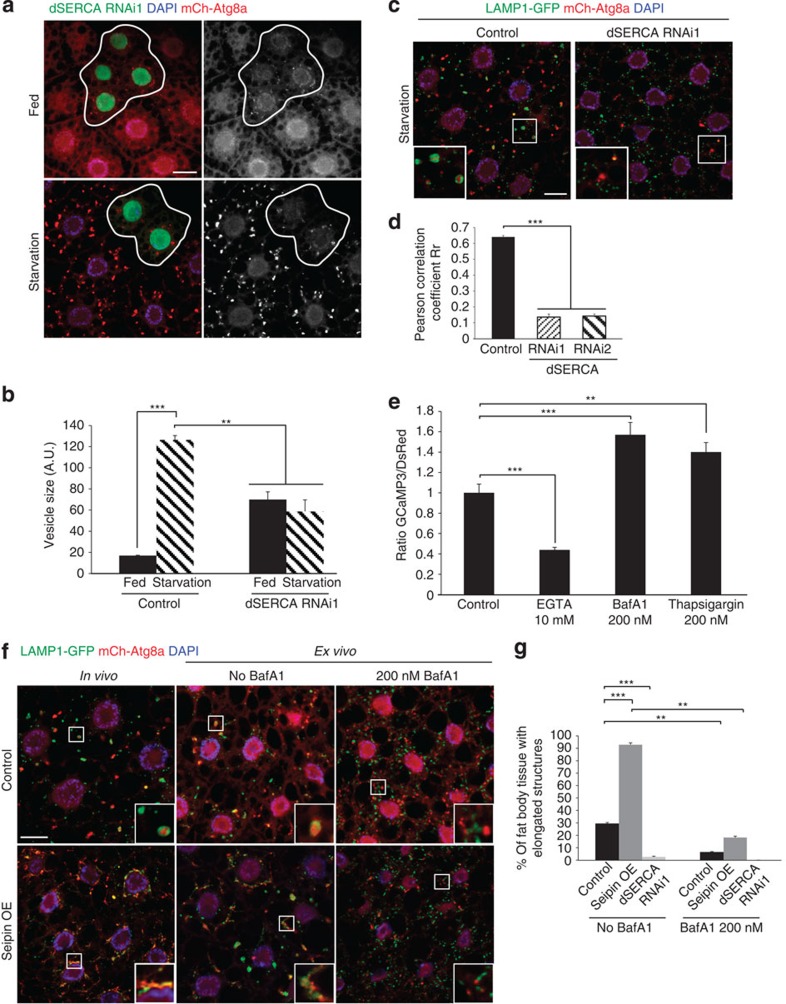
BafA1 blocks autophagosome–lysosome fusion through inhibition of dSERCA. (**a**) Representative confocal images of control (GFP negative) and dSERCA-depleted (GFP-positive clones) fat body cells from well-fed and 4 h starved larvae. Depletion of dSERCA leads to accumulation of mCh-Atg8a punctae (red, and shown in greyscale at right) under fed conditions with no further accumulation in starved samples. (**b**) Quantification of size of Atg8-positive vesicles in control and dSERCA-depleted fat body cells from experiment in **a**. Error bars mark s.e.m. for each genotype (*N*≥5). **P<0.05 and ***P<0.001, Student’s *t*-test. (**c**) Localization of LAMP1-GFP and mCh-Atg8a in control and dSERCA-depleted fat body from 4 h starved larvae. Insets illustrate the presence or absence of autolysosomes. Autolysosomes identified as mCh-Atg8a-positive structures surrounded by LAMP1-GFP were observed only in control. *N*≥9 for each genotype. (**d**) Quantification of co-localization between LAMP1-GFP and mCh-Atg8a. Pearson’s correlation coefficient Rr was calculated using a minimum of nine representative images for each genotype. Error bars mark s.e.m. for each condition. ***P<0.001, Student’s *t*-test. (**e**) Quantification of GCaMP3 fluorescent intensity relative to internal control DsRed. Fat body cells incubated *ex vivo* for 1 h with EGTA (10 mM) showed a significant reduction of GCaMP3/DsRed ratio, while cells treated with BafA1 (200 nM) or thapsigargin (200 nM) presented a strong increase compared with control. Error bars mark s.e.m. for each condition, *N*≥25. **P<0.005, ***P<0.001, Student’s *t*-test. (**f**) Morphology of LAMP1-GFP and mCh-Atg8a structures in control and Seipin-overexpressing cells. Fat body were dissected from larvae either starved for 4 h *in vivo* or incubated for 6 h *ex vivo* in M3 media in the presence or absence of BafA1 (200 nM). In **a**, **c** and **f**, nuclei are labelled with DAPI; blue; scale bars, 10 μm. (**g**) Quantification of the presence of elongated mCh-Atg8a- and LAMP1-GFP-positive tubular structures in fat body cells as shown in **f**. Error bars mark s.e.m. for each condition, *N*≥16. **P<0.05 ***P<0.005, Student’s *t*-test.

We therefore tested the role of the single *Drosophila* SERCA homologue (dSERCA/CaP60A) in autophagic flux and lysosomal fusion. Depletion of dSERCA led to a modest accumulation of mCh-Atg8a-marked vesicles under fed conditions, which was not further increased in response to starvation (Fig. 6a; see Supplementary Fig. 6a for validation of dSERCA depletion). This phenotype was specific to dSERCA, as depletion of other P-type ATPases such as secretory pathway calcium ATPase (SPoCk) or plasma membrane calcium ATPase (PMCA) did not affect the accumulation of mCh-Atg8a-marked vesicles (Supplementary Fig. 6b). dSERCA-depleted cells also failed to show the time-dependent increase in autolysosome size observed in control or V-ATPase-depleted cells (Fig. 6b compare with Fig. 4b), suggesting a defect in autophagosome–lysosome fusion in response to dSERCA depletion. This was supported by a significant reduction in LAMP1-Atg8a co-localization in dSERCA-depleted but not PMCA-depleted cells (Fig. 6c,d and Supplementary Fig. 6c). Thus, genetic silencing of dSERCA gives rise to a defect in autophagosome–lysosome fusion, in agreement with similar effects observed in thapsigargin-treated mammalian cells^38^. Importantly, *ex vivo* incubation of fat body with BafA1 caused a significant increase in cytosolic Ca^2+^ concentration, similar to the effects of thapsigargin (Fig. 6e). Together, these results confirm that the P-ATPase SERCA pump promotes autophagosome–lysosome fusion, and that BafA1 may inhibit fusion in part through its effects on SERCA rather than the V-ATPase.

To further test this idea, we genetically increased dSERCA activity by overexpressing Seipin, a recently identified positive regulator of dSERCA^39^. Seipin was shown to directly bind to dSERCA and promote its Ca^2+^ transport activity in *Drosophila* fat body cells. Interestingly, Seipin overexpression *in vivo* and *ex vivo* induced the formation of elongated Atg8a- and LAMP1-positive tubular structures, suggesting that increased dSERCA activity promotes hyper-fusion and growth of autolysosomes (Fig. 6f). The GFP signal of tandem-tagged Atg8a was quenched in these cells, further indicating successful autophagosome–lysosome fusion (Supplementary Fig. 6d). Treatment of these cells with BafA1 strongly reduced the formation of the tubular Atg8a structures (Fig. 6f,g), consistent with its effects on autophagosome–lysosome fusion and supporting that these effects are mediated in part through a dSERCA-dependent event. To summarize, our results demonstrate that increased activation of dSERCA accelerates autophagosome–lysosome fusion, forming elongated autolysosome-like structures in the cytosol that are inhibited by BafA1. These findings suggest a novel mechanism of action for BafA1, in which inhibition of the P-ATPase dSERCA pump contributes to a block of autophagic vesicle fusion independent of its effects on the V-ATPase and lysosomal pH.

## Discussion

As the end point of the autophagic and endocytic pathways, lysosomes have an especially critical requirement for the low pH generated by V-ATPase activity. The acidic environment of the lysosomal lumen contributes directly to macromolecular breakdown and provides an optimal pH for a wide range of acid hydrolases. In addition, the electrochemical gradient generated by the V-ATPase is used to drive transport of breakdown products such as amino acids out of the lysosome^40^ and to maintain high lysosomal concentrations of Ca^2+^ and other ions^41^. The V-ATPase also plays a critical role in overall lysosomal homeostasis, as acidification is necessary for reformation of lysosomes from the hybrid organelles generated by lysosome–endosome fusion^42^. Consistent with these roles, we find that genetic mutation or depletion of V-ATPase subunits in the *Drosophila* larval fat body disrupts lysosomal acidification and blocks autophagic flux, leading to accumulation of the autophagy substrate Ref(2)P. V-ATPase depletion also leads to loss of Cathepsin activity and formation of enlarged autolysosomes containing multiple single-membrane vesicles, with striking resemblance to the autophagic bodies that accumulate within yeast vacuoles mutant for vacuolar peptidases^29^,^30^. These results indicate that the V-ATPase has an important role in autophagic flux by providing a proper degradative environment in the lysosomal lumen.

The V-ATPase has also been suggested to promote lysosomal fusion with endocytic and autophagic vesicles, as well as synaptic vesicle fusion in neurons and homotypic vacuolar fusion in yeast. The mechanism by which the V-ATPase regulates these membrane fusion events is controversial, with a number of studies demonstrating a purely structural requirement for the membrane-bound V0 complex, which may facilitate formation of a membranous fusion pore and interact directly with components of the fusion machinery such as SNAREs^43^,^44^,^45^. In contrast, other studies have shown that fusion requires vesicle acidity but not the physical presence of the V-ATPase^46^, or that the V-ATPase may regulate fusion by acting as a sensor of vesicular pH^47^. A role for the V-ATPase specifically in autophagosome–lysosome fusion is supported largely by studies with BafA1, which has been shown to cause an arrest of autophagic flux and a separation of autophagosomal and lysosomal markers in multiple cell types^24^,^32^,^48^. Based on the lag time often observed in these experiments, it has been suggested that the effects of BafA1 are secondary to its dissipation of lysosomal acidity^25^, a view also supported by the ability of acidotropic amines such as ammonia and chloroquine to disturb autophagic fusion and flux^48^.

**Figure 7:**
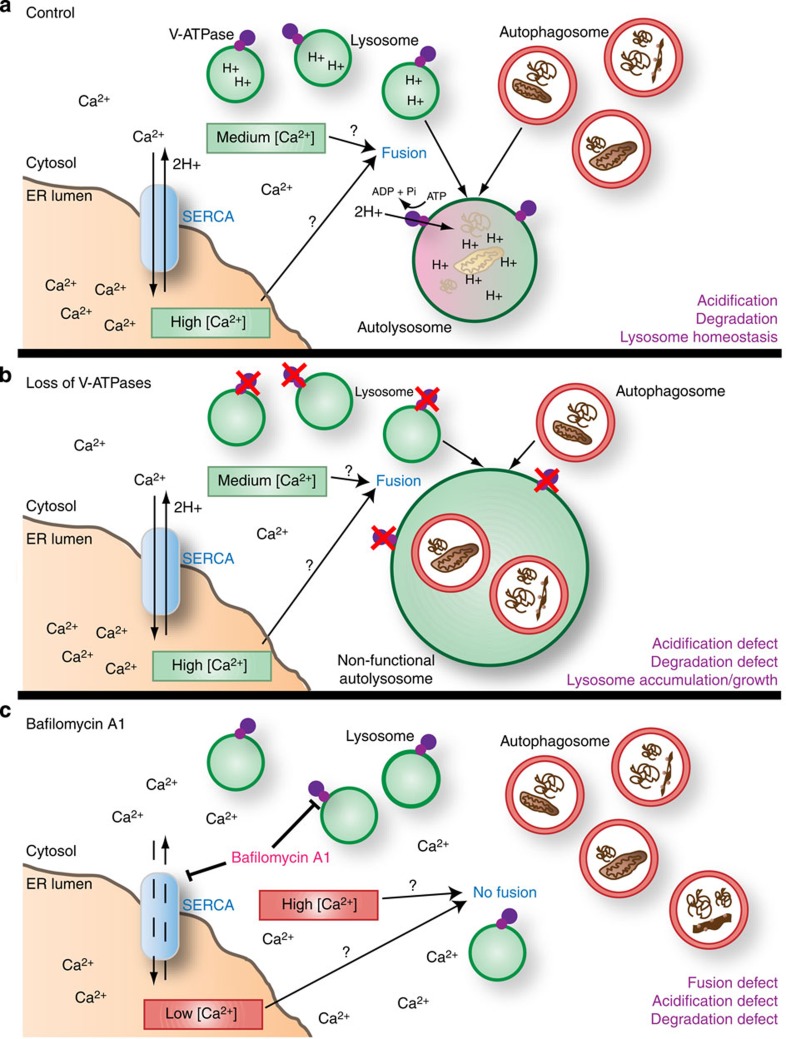
Schematic model of fusion and acidification events. (**a**) Under normal conditions, SERCA generates a Ca^2+^ gradient with a high concentration of Ca^2+^ in the ER lumen. The role of SERCA in autophagosome–lysosome fusion may reflect its ability to maintain a relatively low/moderate cytoplasmic Ca^2+^ concentration, or to allow localized release of ER lumenal Ca^2+^ near sites of fusion. Pumping of protons into the lysosomal lumen by the V-ATPase leads to acidification, activation of hydrolytic enzymes and autophagic cargo degradation. The V-ATPase, along with amino acids released by autophagic breakdown, also promotes activation of mTOR (not shown), which is required for lysosomal reformation. (**b**) Genetic depletion of V-ATPase subunits results in a loss of lysosomal acidification, while Ca^2+^/SERCA-dependent fusion remains active. Continued autophagosome–lysosome fusion in the absence of lysosomal degradation and recycling disrupts lysosomal homeostasis, leading to an expansion of the autolysosomal compartment. (**c**) Inhibition of both the V-ATPase and SERCA by BafA1 blocks lysosomal acidification and dissipates the ER Ca^2+^ gradient. Either abnormally high Ca^2+^ concentration in the cytosol and/or lower Ca^2+^ concentration in the ER lumen may contribute to defective autophagosome–lysosome fusion.

In contrast to these reports, we demonstrate here through multiple independent approaches that genetic loss of any of a number of V-ATPase subunits does not lead to defects in autophagosome–lysosome fusion in the *Drosophila* fat body, despite clear disruption of lysosomal acidity and function. V-ATPase depletion did not disrupt formation of autolysosomes, as identified by co-localization of lysosomal and autophagosomal markers and by ultrastructural analysis. Indeed, these vesicles continued to grow over a 24-h time course of starvation, and this expansion required the function of the autophagosome-specific Q-SNARE Syx17. This finding supports the idea that autophagosomes represent the major vesicular input to lysosomes in these cells under starvation conditions, although our results demonstrate that vesicles from the endosomal and Golgi compartments can also traffic to V-ATPase-depleted lysosomes. Although we cannot exclude that residual partial acidification of lysosomes may contribute to their fusion, our results therefore suggest that neither normal acidification nor a physical presence of the V-ATPase is required for autophagosome–lysosome fusion in these cells (Fig. 7b).

In line with our data, short interfering RNA-mediated depletion of the BafA1 target V0c (ATP6V0C) in human neuroblastoma cells was recently shown to cause a significant decrease in lysosomal acidity, but did not disrupt autophagosome–lysosome fusion^49^. Depletion of V0 subunits also disrupted lysosome function but not fusion in mouse AtT-20 cells^50^. Together, these different outcomes of genetic versus drug-based approaches suggest that BafA1 disrupts autophagosome–lysosome fusion at least in part through a V-ATPase-independent target, several of which have been identified^34^,^51^,^52^.

Here we addressed the idea that the fusion effects of BafA1 reflect its inhibition of the Ca^2+^ ATPase SERCA rather than the V-ATPase. Several previous findings support this possibility: (1) BafA1 has been shown to inhibit SERCA activity *in vitro*^34^; (2) BafA1 increases the levels of cytosolic-free Ca^2+^ in a SERCA-dependent manner in human platelets^53^; (3) inhibition of SERCA by thapsigargin selectively blocks autophagosome–lysosome fusion in mouse embryonic fibroblasts^38^. Notably, both chloroquine and ammonia have also been shown to cause a loss of thapsigargin-sensitive Ca^2+^ stores^54^,^55^. Our findings that the inhibition of autophagosome–lysosome fusion by BafA1 is phenocopied by genetic depletion of dSERCA and that this event is promoted by dSERCA activation strongly support that BafA1 acts through a Ca^2+^-dependent mechanism in this setting.

An essential role for Ca^2+^ in regulating fusion of multiple vesicle types has been long appreciated. Ca^2+^ modulates the activity of several key regulators of fusion including SNAREs, coat proteins and Rab GTPases, and may directly promote membrane destabilization leading to fusion^56^,^57^,^58^. We propose that BafA1 inhibits autophagosome–lysosome fusion by disrupting a critical SERCA-dependent Ca^2+^ gradient required for localized release of Ca^2+^ at sites of fusion (Fig. 7c). Such localized pools of Ca^2+^ could originate from microdomains of the endoplasmic reticulum, which may regulate fusion at lysosome–ER contact sites^59^. The lysosome itself is also a major Ca^2+^ store and may use a SERCA isoform for Ca^2+^ loading^53^. Release of Ca^2+^ from either of these organelles can modulate discharge from the other^59^,^60^,^61^, and the lysosomal Ca^2+^ channel TRPML was recently shown to promote autophagosome–lysosome fusion in *Drosophila*^62^. It is also possible that Ca^2+^ incorporated into the autophagosome during its formation from the ER may promote subsequent fusion events. Depletion of Ca^2+^ from one or more of these sources may disrupt the localized transient release required for fusion. Alternatively, the relevant effect of inhibiting SERCA may be to increase cytosolic Ca^2+^ levels, rather than to decrease lumenal stores. In this regard, high concentrations of Ca^2+^ have been shown to suppress fusion, and drugs that inhibit ER Ca^2+^ channels can alleviate obesity-induced autophagy arrest in mice by promoting autophagosome–lysosome fusion^63^. Indeed, Ca^2+^ has been proposed to play dual roles in regulating autophagy, with modest increases or decreases in Ca^2+^ signalling capable of stimulating autophagy, likely dependent on cellular context^64^. Additional insight into how Ca^2+^ affects individual steps of the autophagy pathway will require a more careful examination of autophagic flux under these different conditions.

In conclusion, different outcomes of genetic versus drug-based studies can provide insights into the relevance of particular molecular targets and functions of chemical compounds, and can help determine which effects of these agents are most relevant to a particular cellular or disease process. Our findings suggest that the effect of BafA1 on lysosomal pH plays a limited role in fusion, and they highlight the need to fully consider potential off-target effects when developing models for disease therapy based on targeting the V-ATPase.

## Methods

### *Drosophila* stocks

Flies were raised at 25 °C on standard cornmeal/molasses/agar media. Transgenic *D. melanogaster* RNAi strains listed in Table 1 and Supplementary Table 1 were obtained from the Bloomington Stock Center (Bloomington, IN) and the Vienna Drosophila RNAi Center (Vienna, Austria). For clonal analysis, these lines were crossed with hs-flp; UAS-Dicer; r4-mCherry-Atg8a Act<CD2<GAL4 UAS-GFPnls^65^. Where indicated, Rab7-GFP^66^ or VhaM8-9-GFP^67^, a gift of Dr M. Boutros (Heidelberg, Germany), was used in place of GFP-nls, or mCherry-Atg8a was omitted to allow visualization of other markers. For GAL4-mediated expression throughout the fat body, the Cg-GAL4 driver was used in conjunction with UAS-mCherry-Atg8a, UAS-LAMP1-GFP^68^, UAS-GFP-mCherry-Atg8a (ref. 17) or UAS-GFP-Ref(2)P (ref. 20) markers. UAS-Seipin^39^ was kindly shared by Dr X. Huang (Beijing, China). UAS-DsRed; P(20xUAS-GCaMP3)attP^69^ flies were kindly shared by Dr Myung Jun Kim (Minneapolis, MN). Flp-mediated V-ATPase loss of function clones were induced using FRT-linked mutants from the Bloomington Stock Center and Drosophila Genetic Resource Center (Kyoto, Japan) through 1 h heat-shock at 37 °C immediately after short collection of embryos^20^.

### Histology and imaging

Staged 72 h larvae expressing transgenic fluorescent markers were transferred to vials containing fresh fly food for 24 h, then either kept in food (fed condition) or starved for the indicated times in 20% sucrose in PBS. The larvae were then bisected, inverted and fixed in 3.7% formaldehyde at 4 °C overnight on orbital. After washing three times for 15 min in PBS with 0.1% Triton X-100 (PBS-T) and counterstaining with 1 μg ml^−1^ 4,6-diamidino-2-phenylindole, the fat bodies were dissected out and mounted in Vectashield (Vector Laboratories, Burlingame, CA). Larval samples were imaged at room temperature on a Zeiss LSM710 confocal microscope equipped with a × 40 (W) objective lens (APO DIC III numerical aperture 1.2) and acquired using Zeiss software Zen 2010. Laser lines used in this study were 405, 488 and 561 nm. Red/green/blue (RGB) and greyscale images were further processed with ImageJ (National Institutes of Health, USA) or Photoshop CS3 and assembled into figures using Adobe Illustrator CS3 (San Jose, CA).

To monitor Cathepsin B activity or lysosomal acidification, third instar larvae were bisected and carcasses were incubated for 3 min in PBS containing 1 × Magic Red cresyl violet-(RR)2 (Immunochemistry Technology, Bloomington, MN) or LysoTrackerRed (Invitrogen), respectively, and imaged immediately on a Zeiss Axioscope-2 microscope^15^. For TR-avidin uptake assay, 2 h starved (in 20% sucrose) or fed larvae were bisected and inverted in PBS, and then transferred to a microfuge tube containing 80 μg ml^−1^ TR-avidin (Invitrogen) in M3 insect medium (Sigma-Aldrich) containing 5% fetal calf serum and 1 × insect medium supplement (Sigma-Aldrich). Human insulin (10 μg ml^−1^,Sigma-Aldrich) was added to media to mimic fed condition. Carcasses were incubated for 2 h at RT with rocking, rinsed twice with ice-cold PBS and washed three times for 5 min with ice-cold PBS at 4 °C, and then fixed and mounted in Vectashield as described above^70^.Indirect immunofluorescence labelling of endogenous Ref(2)P was performed in clones using GAL4/UAS system in the larval fat body. After fixation overnight in 3.7% paraformaldehyde, carcasses from third instar larvae were washed for 2 h in PBS, permeabilized for 15 min in PBTX-DOC (PBS with 0.1% Triton X-100 and 0.05% sodium deoxycholate) and blocked for 3 h in 3% goat serum in PBTX-DOC. Incubation with primary antibody rabbit polyclonal anti-p62 (1:2,000) in 1% goat serum in PBTX-DOC was overnight at 4 °C with rocking. After three washes of 30 min each in PBTX-DOC, samples were incubated with secondary antibody goat anti-rabbit Alexa 568 (1:1,500; Invitrogen) in 1% goat serum in PBTX-DOC for 4 h at RT. After three washes of 15 min in PBTX-DOC and 1 × 10 min in PBS, fat bodies were mounted in 50% glycerol/PBS with 0.2 μM 4,6-diamidino-2-phenylindole (DAPI). Images were captured on a Zeiss Axioimager M2 microscope equipped with an Apotome2 grid confocal unit, a Plan-NeoFluar 40 × 0.75 numerical aperture objective, Axiocam Mrm camera and Axiovision software using a MinMax setting for automatically adjusting image levels^22^.

### Image analysis

Co-localization of cytoplasmic LAMP1-GFP and mCherry-Atg8a punctae was analysed in ImageJ (NIH), using the Intensity Correlation Analysis plugin to determine Pearson’s correlation coefficient of split red and green channels (15 confocal images per sample). Numbers of mCherry-GFP-Atg8a autophagic vesicles were assessed using the Adobe Photoshop CS3 measurement analysis option; acidified (red only) vesicles were visualized by subtraction of the green channel from the initial RGB images. Diameters of 10 randomly selected mCh-Atg8a-positive vesicles per cell were measured using ImageJ.

### Intracellular Ca^2+^ concentration measurement

Freshly dissected carcasses of third instar larvae expressing DsRed and GCaMP3 driven by Cg-GAL4 were incubated *ex vivo* with rocking in M3 medium (S3652, Sigma-Aldrich) containing 10 μg ml^−1^ human insulin (I9278, Sigma-Aldrich) for 1 h at room temperature in the presence of 10 mM EGTA, 200 nM BafA1 (B1793, Sigma-Aldrich) or 200 nM thapsigargin (T9033, Sigma-Aldrich). Fat bodies were mounted in 1 × PBS and GFP/DsRed fluorescence ratios were measured under identical settings using a Zeiss LSM710 confocal microscope as described above.

### TEM

Larvae were dissected and fixed overnight at 4 °C using a solution of 3.2% paraformaldehyde, 0.5% glutaraldehyde, 1% sucrose and 0.028% CaCl_2_ in 0.1 N sodium cacodylate, pH 7.4, followed by postfixation in 0.5% osmium tetroxide for 1 h and embedding into Durcupan (Fluka). Sections (70 nm) were stained in Reynold’s lead citrate and photographed on a transmission electron microscope (JEM-1011; JEOL and Olympus Morada camera) using iTEM software (Olympus). A total of 15 randomly taken low-magnification images of sections from three animals were evaluated per genotype by manually encircling relevant structures in Photoshop and calculating their percentage of area relative to total cytoplasm.

### Reverse transcriptase–PCR

Reverse transcriptase–PCR was carried out by preparing RNA from larval fat bodies dissected from 20–25 L3 stage larvae per genotype using Purelink RNA Mini Kit (Ambion), followed by cDNA synthesis using Revertaid First Strand cDNA synthesis Kit (Fermentas) and amplification with DreamTaq Green (Fermentas) for 25 PCR cycles in a Swift Maxi thermocycler (ESCO). The following primers were used: VhaSFD (5′-ATCTATTCCATGACACAGCCG-3′ and 5′-CAGGATGGATAGCTGCTTCAG-3′), Vha16-1 (5′-CCTTCTTCGGAGTTATGGGAG-3′ and 5′-AGCCGTAGAGACCCAACACTT-3′) and SERCA (5′-GTCCCTCAACTTCTTCGGAAC-3′ and 5′-ATCTTGTCACCGACAGACACC-3′).

## Additional information

**How to cite this article:** Mauvezin, C. *et al*. Autophagosome–lysosome fusion is independent of V-ATPase-mediated acidification. *Nat. Commun*. 6:7007 doi: 10.1038/ncomms8007 (2015).

## Supplementary Material

Supplementary Information Supplementary Figures 1-6 and Supplementary Tables 1-2

## Figures and Tables

**Table 1:** Disruption of *Drosophila* V-ATPase subunits.

***Drosophila*** **gene**	**Subunit**	**RNAi constructs and genotypes affecting autolysosome size and number**
*V1*		
Vha68-2	V1A	*P{KK108767}v110600* *
		*TRiP HMS01056* *
		*Vha68-2[EP2364] FRT40* *
Vha44	V1C	*P{KK108970}v101527* †
Vha36-1	V1D	*P{KK108864}v110078*
Vha26	V1E	*P{KK109326}v102378* ‡
Vha14-1	V1F	*P{KK102097}110160*
VhaSFD	V1H	*P{GD8795}v47471*
		*VhaSFD[EY04644] FRT40* *
		
*V0*		
Vha16-1	V0c	*P{KK109332}v104490* †
VhaPPA1-1	V0c''	*P{GD16478}v47187* *
VhaAC39-1	V0d	*TRiP HMS01442* *
		*P{GD9859}v20950* *
		*FRT19 VhaAC39-1 FZ29* *
		
*Accessory*		
VhaAC45	V1S1	*P{KK105641}v101726* †
		*TRiP HMS01717* *
VhaM8-9	PRR	*FRT82 VhaM8-9[LL04700]*

RNAi, RNA interference.

List of V-ATPase subunits whose depletion or mutation resulted in accumulation and enlargement of autolysosomes in larval fat body cells. Phenotypes were observed in response to both clonal and uniform depletion unless otherwise noted.

^*^Additional phenotype of strong cell size reduction.

^†^Additional phenotype of moderate cell size reduction.

^‡^Autolysosome phenotype observed only in response to clonal depletion.
