# Supplementary material for: Autophagosome–lysosome fusion is independent of V-ATPase-mediated acidification
Source: Nat Commun. 2015 May 11;6:7007. doi: 10.1038/ncomms8007 (PMC4428688; doi:10.1038/ncomms8007)
Supplement: Supplementary Information — Supplementary Figures 1-6 and Supplementary Tables 1-2 [file ncomms8007-s1.pdf]

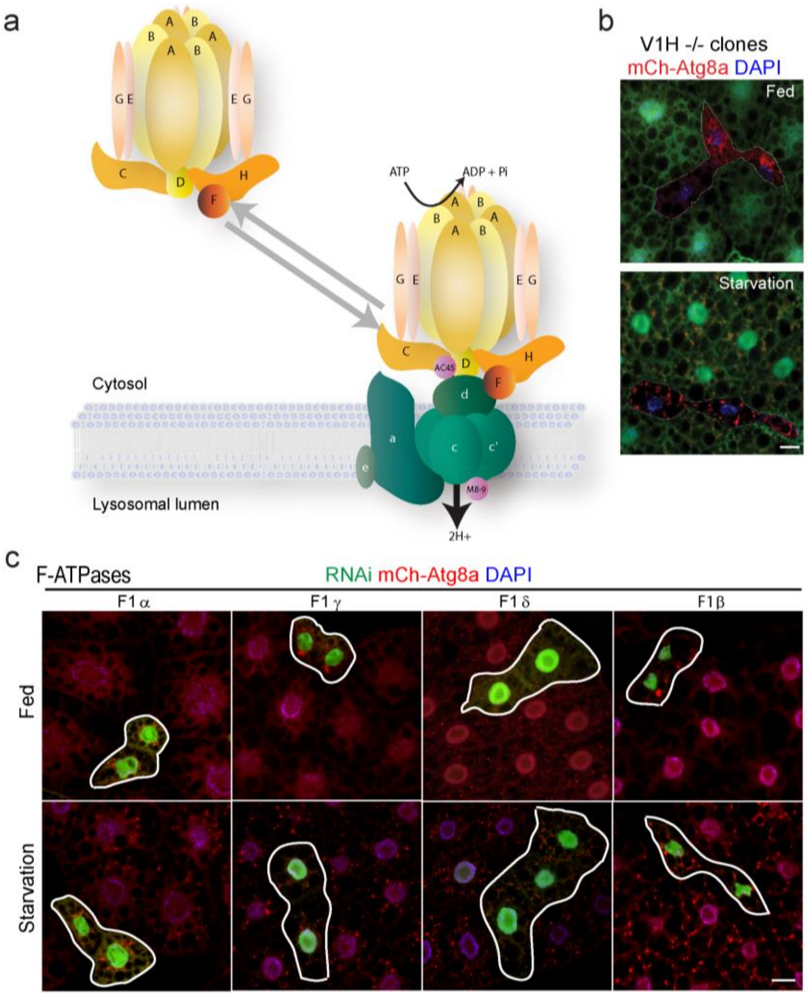

Supplementary Fig. 1 V-ATPase depletion induces unique and robust phenotype in Drosophila fat body cells.

a. Schematic of the V-ATPase proton pump macro-complex structure. The V1 complex is composed of eight subunits and docks to the membrane-bound V0 complex (six subunits) for proton pump activation. For each ATP molecule hydrolyzed, two protons enter the lysosomal lumen. **b**. Autophagic vesicles accumulate in V1H loss of function mutant cells. All cell express mCh-Atg8a (red), which localizes to autophagosomes/autolysosomes in starved wild-type cells (GFP-positive). Cells clones that are homozygous mutant for V1H (GFP-negative) accumulate enlarged mCh-Atg8a punctae under both fed and starved conditions. Nuclei are stained with DAPI and detected in blue. Images are representative of 10 larvae per condition. Scale bars, 10 µm. **c**. Representative confocal images of fat body cells expressing transgenic RNAi constructs that target F-ATPase subunits (GFP-positive clones) in well fed and 4 h starved animals. All cell express mCh-Atg8a (red). F-ATPase-depleted cells abnormally accumulate autophagic structures in their cytosol under fed conditions. No further increase in size or number of Atg8a-positive structures is detectable after 4 h starvation. N≥8 larvae for each genotype and condition. Nuclei are stained with DAPI (blue). Scale bars, 10 µm.

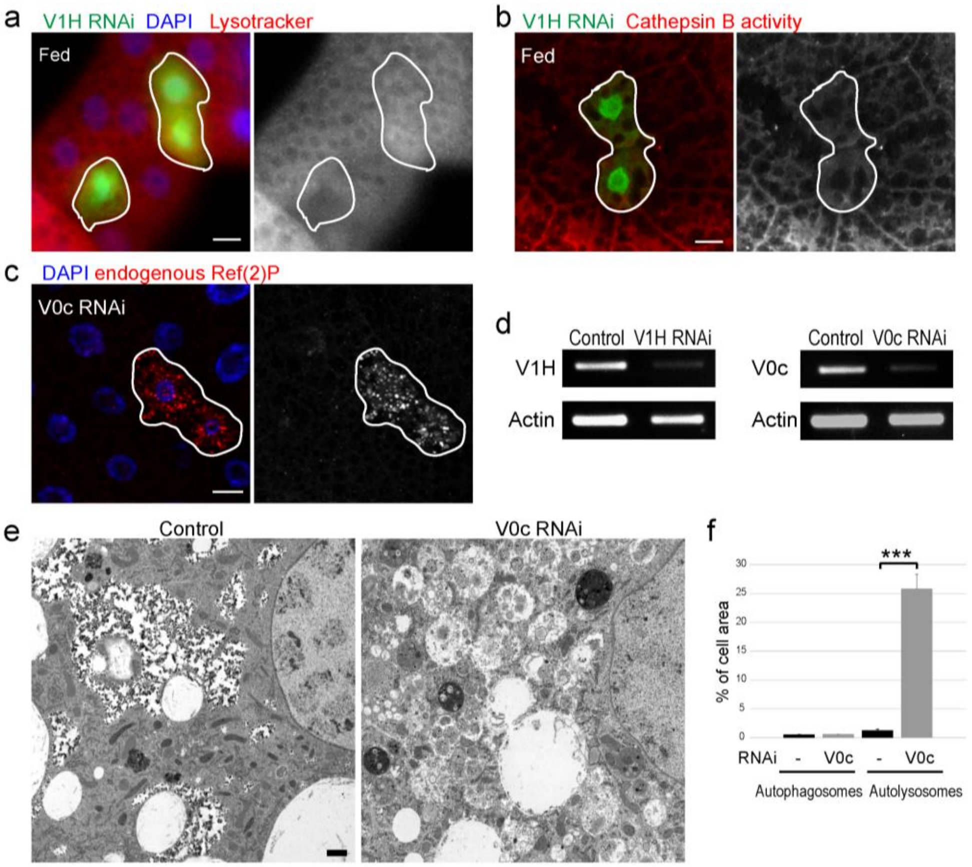

Supplementary Fig. 2. Loss of V-ATPase causes an accumulation of non-functional autolysosomes.

a. Punctate LysoTracker Red staining is not observed in V1H-depleted cells (GFP-positive) or in adjacent wild type cells under nutrient-rich conditions (N≥8). LysoTracker signal alone is shown in the grayscale image at right. **b**. Fluorescence images of fat body cells stained for Cathepsin B enzyme activity with Magic Red cresyl violet-(RR)2 in nutrient-rich conditions (N≥6). No punctate staining is observed in either control cells (GFP-negative) or V1H-depleted cells (GFP-positive). Magic Red signal alone is shown in grayscale. **c**. Depletion of the V0c subunit (RNAi-expressing clone is outlined) leads to accumulation of endogenous Ref(2)P under nutrient-rich conditions. Ref(2)P signal alone is shown in grayscale. Scale bars, 10 μm. In panels a and c, nuclei are stained with DAPI in blue. **d**. Efficiency of RNAi-mediated depletion of V1H and V0c mRNA was tested by RT-PCR. Actin mRNA levels were used as internal control. **e**. TEM images of control (*w1119*) and V0c-depleted (*Cg-GAL4/UAS-V0c-dsRNA*) fat body cells under starved conditions (4 h). Scale bar, 5 μm. **f**. Quantification of percent cell area occupied by double-membrane autophagosomes and single-membrane autolysosomes in control and V0c-depleted cells as displayed in panel e. Error bars mark standard error of the mean (SEM), N=3 animals, 15 images for each genotype. *** $p<0.001$, Student’s t-test.

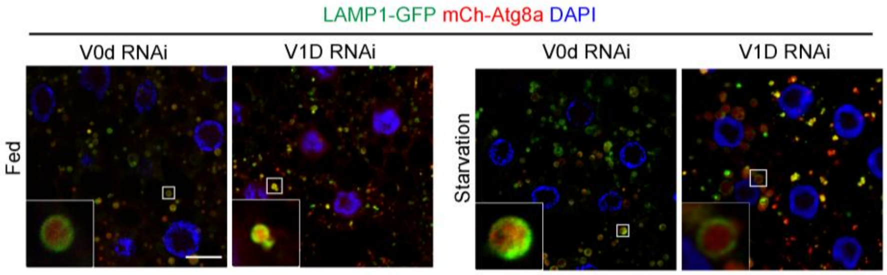

Supplementary Fig. 3. Autophagosome-lysosome fusion occurs in cells depleted for V-ATPase.

Confocal images of fat body cells co-expressing mCh-Atg8a (red) and the lysosomal marker LAMP1-GFP (green) and RNAi against V-ATPase subunits V0d or V1D under fed or 4 h starvation conditions (N≥6). Insets illustrate the presence of autolysosomes representing fusion between autophagosomes and lysosomes. Scale bar represents 10 µm. Nuclei are labeled with DAPI (blue).

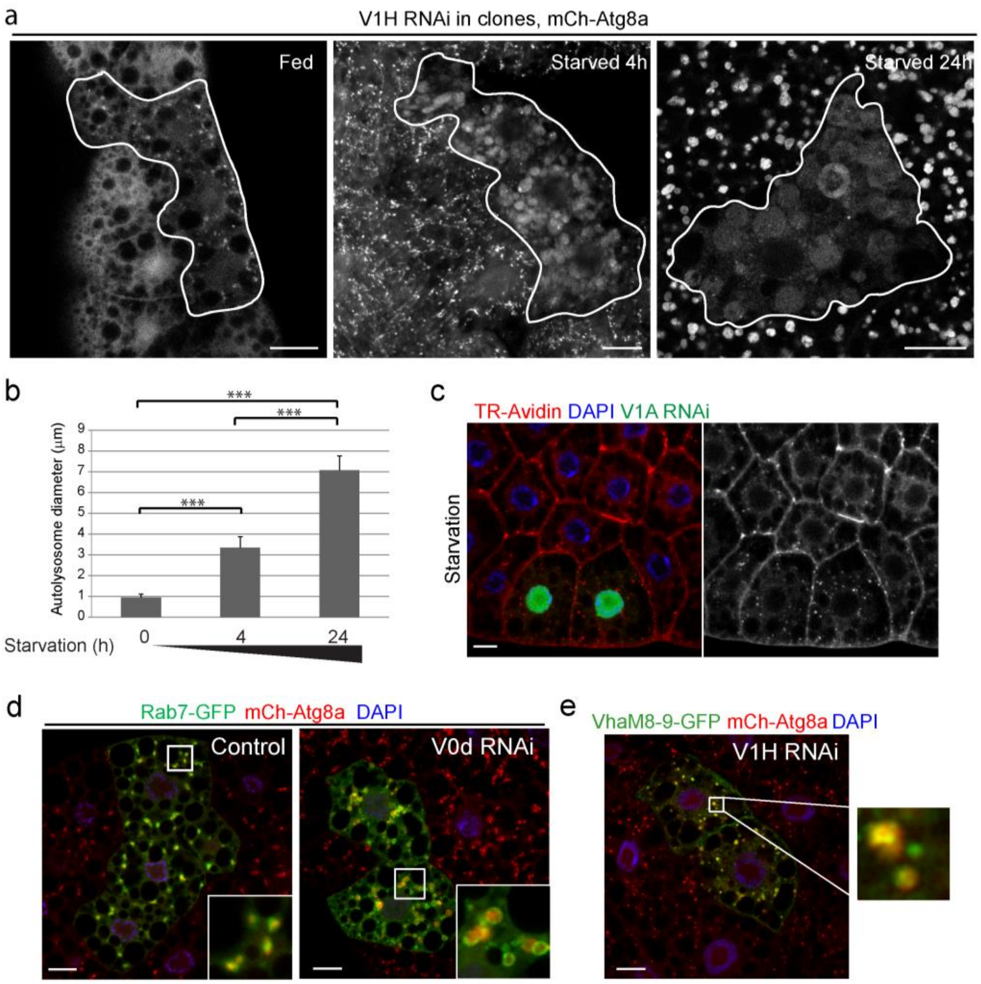

Supplementary Fig. 4. **Autophagic vesicles expand and fuse with vesicles emerging from endocytic pathway or from Golgi in cells depleted for V-ATPase.**

a. Autophagic vesicles expand in V-ATPase-depleted cells in response to prolonged starvation. V1H was depleted in GFP-marked clones (clonal boundaries are outlined) within fat bodies expressing mCh-Atg8a shown here in grayscale. **b**. Quantification of mCh-Atg8a-positive vesicle diameter in V1H-depleted cell clones of 0, 4 h and 24 h starved larvae as shown in panel b. Error bars mark SEM; N=8 biological samples for each condition. *** $p<0.001$, Student's t-test. **c**. Representative image of fat body incubated for 2 h with the endocytic tracer Texas Red-Avidin (red, shown in grayscale at right). Tracer uptake is evident both in control cells and in V1A-depleted cells (GFP-positive clone) (N≥7). **d**. Confocal images of fat body cells with uniform expression of mCh-Atg8a and clonal co-expression of Rab7-GFP and V0d RNAi (N≥5 for each genotype). Rab7-GFP colocalizes with mCh-Atg8a both in control and V0d-depleted clones. **e**. Colocalization of mCh-Atg8a and the V0 subunit marker VhaM8-9-GFP (green) which marks V1H-depleted clones (N≥9). In all panels, scale bar represents 10 µm. Nuclei are labeled with DAPI (blue) in panels c-e.

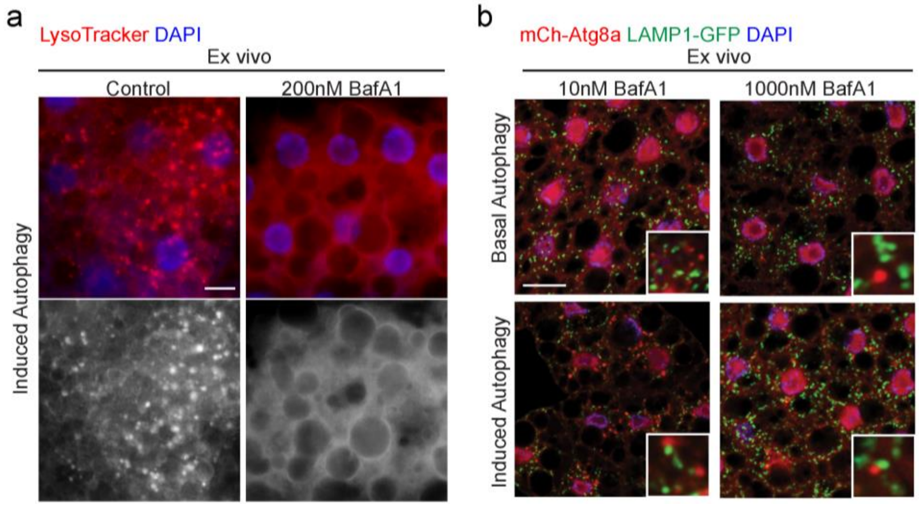

Supplementary Fig. 5. BafilomycinA1 blocks vesicles acidification and autophagosome-lysosome fusion in Drosophila fat body cells.

a. LysoTracker Red staining in wild type fat body tissues incubated 6 h ex vivo in the absence (Control) or presence of 200 nM BafilomcyinA1. Autophagy was induced by the omission of insulin during incubation. LysoTracker signal alone is shown in the grayscale image below. N≥7 for each condition. **b**. Representative images of fat body cells co-expressing LAMP1-GFP and mCh-Atg8a incubated ex vivo for 6 h with 10 nM or 1 µM BafilomycinA1 in the presence (basal autophagy) or absence (induced autophagy) of 10 µg/ml insulin (N≥10). Genotype: *Cg-Gal4 UAS-mCherry-Atg8a UAS-LAMP1-GFP/+*. In all panels, scale bar represents 10 µm. Nuclei are labeled with DAPI (blue).

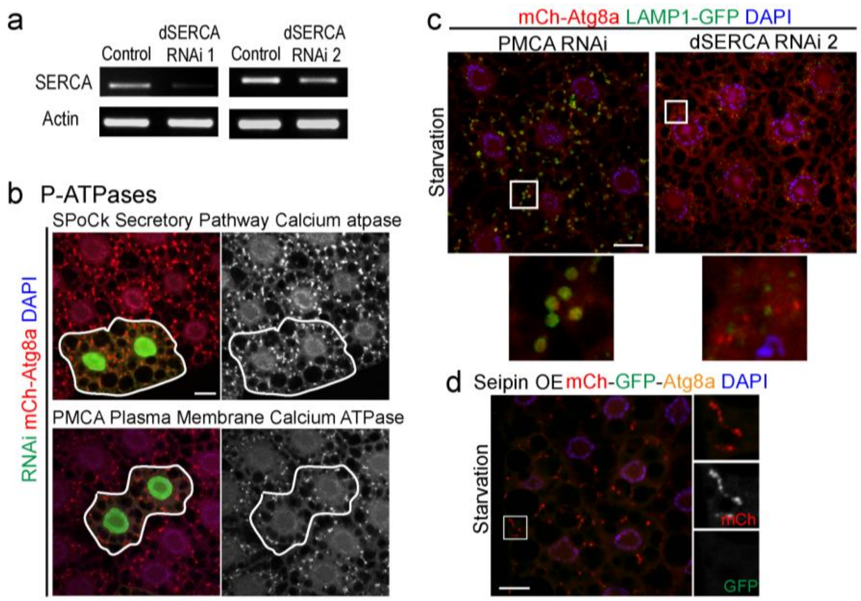

Supplementary Fig. 6. dSERCA activity is necessary for autophagosome-lysosome fusion.

a. Efficiency of the two RNAi knock-down for dSERCA was tested by RT-PCR. Actin levels were used as internal control. **b**. SPoCk- or PMCA-depleted cells (GFP+ clones) show normal induction and size of mCh-Atg8a punctae (red, grayscale images at right) in 4 h starved larval fat body tissue. N≥6 for each genotype. **c**. Co-localization of LAMP1-GFP and mCh-Atg8a is normal in fat body cells depleted of PMCA and defective in cells depleted of dSERCA (RNAi2). N≥6 for each genotype. **d**. Representative confocal image of elongated mCh-GFP-Atg8a vesicles in fat body cells overexpressing Seipin under 4 h starvation conditions (N≥15). The quenching of GFP signal indicates successful acidification and fusion of lysosomes with Atg8a-positive autophagosomes. In panels b to d, nuclei are labeled with DAPI (blue); scale bars, 10 μm.

Drosophila gene	CG Code	Subunit	Function
V1 ATPase			
Vha68-1	CG12403	V1A	Catalytic
Vha68-2	CG3762		
Vha68-3	CG5075		
Vha55	CG17369	V1B	Catalytic
Vha44	CG8048	V1C	Regulatory
Vha36-1	CG8186	V1D	Central rotor axle
Vha36-2	CG13167		
Vha36-3	CG8310		
Vha26	CG1088	V1E	Stator
Vha14-1	CG8210	V1F	Central rotor axle
Vha14-2	CG1076		
Vha13	CG6213	V1G	Stator
VhaSFD	CG17332	V1H	Regulatory
V0 ATPase			
Vha100-1	CG1709	V0a	Stator/Proton transport
Vha100-2	CG7679		
Vha100-3	CG30329		
Vha100-4	CG7678		
Vha100-5	CG12602		
Vha16-1	CG3161	V0c'	Rotor
Vha16-2	CG32089		
Vha16-3	CG32090		
Vha16-4	CG9013		
Vha16-5	CG6737	V0c'	Proton Transport/Rotor
VhaPPA1-1	CG7007	V0c''	Proton Transport/Rotor
VhaPPA1-2	CG7026		
VhaAC39-1	CG2934	V0d	Coupling/Rotor
VhaAC39-2	CG4624		
VhaM9.7-1	CG1268	V0e	
VhaM9.7-2	CG7625		
VhaM9.7-3	CG11589		
VhaM9.7-4	CG14909		
V-ATPase Accessory subunits			
VhaAC45	CG8029	AC-45	Accessory
VhaM8-9	CG8444	M8-9	Accessory

Supplementary Table 1. **Drosophila V-ATPase subunits**

Drosophila gene	Subunit	Genotype
V1 ATPase		
Vha68-1	V1A	*Vha68-1[EY02923] FRT40A*
		P{KK106019}v108701
Vha68-3	V1A	*P{GD11527}v41646*
Vha55	V1B	*P{GD9363}v46554*
Vha44	V1C	*TRiP HMS00821*
		FRT42D Vha44[KG00915]
		FRT42D Vha44[KG07119]
Vha36-1	V1D	*FRT42D Vha36[k07207]*
Vha36-2		*P{KK104055}v104111*
Vha36-3		*P{KK112552}v104048*
Vha14-2	V1F	*P{KK111776}v102478*
VhaSFD	V1H	*TRiP HMS02144*
Vha13	V1G	*P{KK112098}v106536*
V0 ATPase		
Vha100-1	V0a	*P{KK105748}v1089905*
		TRiP JF02059[a]
Vha100-2		*P{KK103346}v109763*
Vha100-3		*P{KK104744}v110051*
Vha100-5		*P{KK106387}v106611*
		TRiP HM04032
Vha16-2	V0c	*P{KK111843}v106520*
Vha16-3		*P{KK110584}v102067*
Vha16-5		*P{KK110979}v107481*
		TRiP JF01821
VhaPPA1-1	V0c''	*FRT82B VhaPPA1-1 [LL03165]*
VhaPPA1-2		*P{GD17023}v48830*
VhaAC39-2	V0d	*P{GD10714}v34303*
VhaM9-7a	V0e	*P{KK107389}v104315*
VhaM9-7b		*P{GD3898}v30384*

VhaM9-7c	V0e	*TRiP JF02029*
		P{KK103881}v101574
VhaM9-7d		*P{KK112066}v106115*
V-ATPase Accessory subunits		
VhaM8-9	PRR	*P{KK107676}v105281*
Drosophila name	Subunit	Genotype
F-ATPases		
bellweather	F1α	*TRiP JF02896*[a,b]
ATPsyn-beta	F1β	*TRiP JF02792*[a,b]
		TRiP JF02892[a,b]
ATPsyn-d	F1δ	*TRiP HMS01078*
ATPsyn-gamma	F1γ	*TRiP JF03150*[a,b]

Drosophila name	Type	Genotype
P-ATPases		
Ca-P60A/SERCA	IIA	*TRiP JF01948 (RNAi 1)*[c]
		TRiP HMS02878 (RNAi 2)[c]
SPoCk	IIA	*TRiP JF02988*
PMCA	IIB	*TRiP JF01145*

Drosophila name	Type	Genotype
Cathepsins		
CG4572	Cathepsin A	*P{KK109136}v101429*
Cathepsin B1	Cathepsin B	*P{KK102677}v108315*
		TRiP HM05090
		TRiP HMS00906
Cathepsin D	Cathepsin D	*TRiP HM05189*
		P{GD5487}v31012
CG12163	Cathepsin F	*P{KK100598}v107589*
26-29KD proteinase	Cathepsin F	*P{KK103643}v100102*
CG30088	Cathepsin G	*P{KK111807}v106412*
Cysteine proteinase-1	Cathepsin L	*P{KK107765}v110619*
		TRiP HMS00725
CG11459	Cathepsin L	*P{KK111883}v102522*

Drosophila name		Genotype
Miscellaneous		
Syntaxin17		*TRiP JF01937*[d]

Supplementary Table 2. **Additional RNAi and mutant lines used in this study**. Listed are genes whose depletion or mutation did not result in abnormal accumulation and enlargement of autolysosomes in larval fat body cells, unless otherwise noted. [a] Accumulation of Atg8-positive punctae under fed conditions; no phenotype under starvation conditions. [b] Cell size reduction. [c] Accumulation of Atg8-positive punctae under fed conditions; reduced induction under starvation conditions. [d] Accumulation of Atg8-positive structures in fed and starved animals and disruption of autophagosome-lysosome fusion.
